# Casein kinase 1α decreases β-catenin levels at adherens junctions to facilitate wound closure in *Drosophila* larvae

**DOI:** 10.1242/dev.175133

**Published:** 2019-10-15

**Authors:** Chang-Ru Tsai, Michael J. Galko

**Affiliations:** 1 Program in Developmental Biology, Baylor College of Medicine, Houston, TX 77030, USA; 2 Department of Genetics, University of Texas MD Anderson Cancer Center, Houston, TX 77030, USA; 3 Genetics & Epigenetics Graduate Program, University of Texas MD Anderson Cancer Center, Houston, TX 77030, USA

**Keywords:** Wound repair, Adherens junctions, Epithelium, Casein kinase 1α, β-Catenin, *Drosophila*

## Abstract

Skin wound repair is essential to restore barrier function and prevent infection after tissue damage. Wound-edge epidermal cells migrate as a sheet to close the wound. However, it is still unclear how cell-cell junctions are regulated during wound closure (WC). To study this, we examined adherens junctions during WC in *Drosophila* larvae. β-Catenin is reduced at the lateral cell-cell junctions of wound-edge epidermal cells in the early healing stages. Destruction complex components, including Ck1α, GSK3β and β-TrCP, suppress β-catenin levels in the larval epidermis. Tissue-specific RNAi targeting these genes also caused severe WC defects. The *Ck1α^RNAi^*-induced WC defect is related to adherens junctions because loss of either β-catenin or E-cadherin significantly rescued this WC defect. In contrast, *TCF^RNAi^* does not rescue the *Ck1α^RNAi^*-induced WC defect, suggesting that Wnt signaling is not related to this defect. Direct overexpression of β-catenin recapitulates most of the features of Ck1α reduction during wounding. Finally, loss of Ck1α also blocked junctional E-cadherin reduction around the wound. Our results suggest that Ck1α and the destruction complex locally regulate cell adhesion to facilitate efficient wound repair.

## Abstract (precis)

**Summary:** Reduction of the adherens junction component β-catenin is a crucial mechanism for regulating the cell-cell interactions that ensure rapid epidermal wound closure in *Drosophila* larvae.

## INTRODUCTION

*Drosophila* studies in wound repair and regeneration have provided many insights at the molecular and cellular levels (Nakamura et al., 2018; Tsai et al., 2018; Zulueta-Coarasa and Fernandez-Gonzalez, 2017). Different signaling pathways and actin regulators play crucial roles during this process (Tsai et al., 2018). To close a wound gap, the epidermal cells become motile but at the same time maintain their adhesive contacts with their neighbors. How cells balance motility with adhesion is not yet clear.

Adherens junctions (AJs) are calcium-dependent adhesion complexes that are important for holding cells together within diverse epithelial tissues (Pinheiro and Bellaïche, 2018). Analysis of AJ function *in vivo* during vertebrate wound healing is complicated by the fact that loss of cadherins is generally lethal (Larue et al., 1994; Riethmacher et al., 1995). During fly embryonic wound healing, AJ components, including E-cadherin, β-catenin and α-catenin, are redistributed around the wound margin (Abreu-Blanco et al., 2013; Hunter et al., 2015; Matsubayashi et al., 2015; Wood et al., 2002; Zulueta-Coarasa et al., 2014). Specifically, AJ components are decreased at the interfaces between wound-edge cells and increased at the wound-edge cellular junctions where these cells are joined (Hunter et al., 2015; Matsubayashi et al., 2015). E-cadherin levels around embryonic wounds are negatively regulated by endocytosis (Hunter et al., 2015), which is in turn required for normal healing (Hunter et al., 2015; Matsubayashi et al., 2015). E-cadherin overexpression delays WC and reduces actin protrusions (Hunter et al., 2015). E-cadherin is also regulated transcriptionally by Toll/NFκB signaling (Carvalho et al., 2014). Whether regulation of AJ levels and function are important in post-embryonic healing, which employs directed cell migration over contraction of an actin cable (Tsai et al., 2018), is not yet clear.

In addition to its essential role at the adherens junction, β-catenin is also the downstream transcriptional co-activator of Wnt/Wingless signaling. Wnt signaling regulates regenerative repair in *Drosophila* imaginal discs (Hariharan and Serras, 2017; Schubiger et al., 2010; Smith-Bolton et al., 2009). In the absence of Wnt ligand, cytoplasmic β-catenin is phosphorylated and ubiquitylated by a protein complex termed the β-catenin destruction complex. This complex consists of casein kinase 1 alpha (Ck1α), glycogen synthase kinase 3β (GSK3β), Axin, adenomatous polyposis coli (APC), protein phosphatase 2A (PP2A) and the E3-ubiquitin ligase β-TrCP. Within the β-catenin destruction complex, Ck1α phosphorylates β-catenin and promotes its degradation (Yanagawa et al., 2002; Zhang et al., 2006). Ubiquitylated β-catenin is subsequently degraded by the proteasome (Aberle et al., 1997). Upon Wnt ligand-receptor binding, β-catenin is released from the destruction complex and translocates to the nucleus where it binds to the transcription factor, T cell factor (TCF or Pangolin in *Drosophila*), to activate downstream gene expression (Hecht and Kemler, 2000). The role of Wnt signaling and the destruction complex in larval WC have not been tested to date.

We investigated the role of β-catenin during larval epidermal WC and found that junctional β-catenin is locally reduced after wounding on membranes radial to the wound site. We further showed that the β-catenin destruction complex is crucial to downregulate junctional β-catenin and facilitate WC. The *Ck1α^RNAi^*-induced WC defects are related to its functions at the adherens junctions but not to roles in Wnt signaling.

## RESULTS

### Reduction of junctional β-catenin in wound-edge epidermal cells

**Fig. 1.**
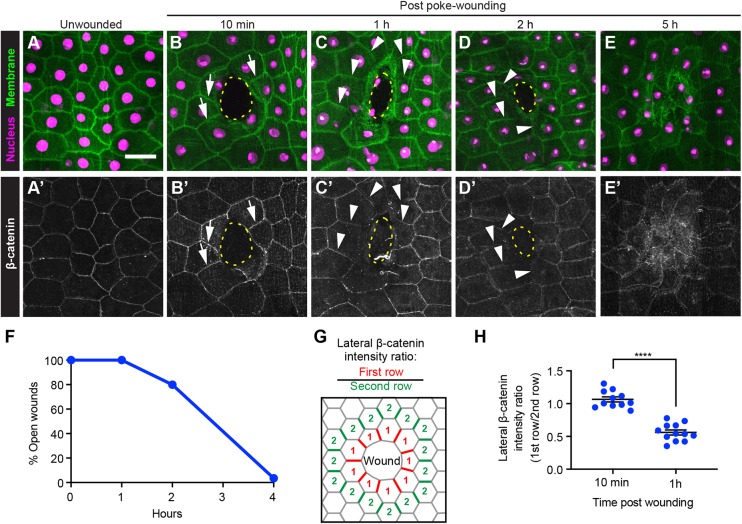
**Junctional β-catenin in wound-edge epidermal cells is reduced after wounding.** (A-E′) Dissected epidermal whole mounts of unwounded (A,A′) or poke-wounded (B-E′) third instar larvae expressing *UAS-DsRed2nuc* (nuclei, magenta) and *UAS-src-GFP* (cell membranes, green) via the *A58-Gal4* driver 10 min (B,B′), 1 h (C,C′), 2 h (D,D′) and 5 h (E,E′) after wounding. (A-E) The nuclei and cell membrane. (A′-E′) The adherens junctions of the same samples immunostained using anti-β-catenin antibodies (white). Scale bar: 50 μm. Dotted yellow lines indicate wound borders. Arrows in B,B′ highlight examples of clear junctional β-catenin signal (B′) and membrane-GFP signal (B). Arrowheads in C-D′ highlight examples of reduced junctional β-catenin (C′,D′) where membrane-GFP is still present (C,D). (F) Quantitation of open poke wounds in control larvae. The epidermal reporter used was *e22c-Gal4, UAS-LifeAct-Cherry, UAS-luciferase^RNAi^*. *n*≥20 for each time point. (G) Schematic of quantitation strategy for measuring β-catenin levels on lateral segments near the wound. (H) Quantitation of junctional β-catenin ratio in first- and second-row wound-edge epidermal cells. Each dot represents one larva. Data are mean±s.e.m. ****P<0.0001 (unpaired *t*-test).

To examine AJs during wound healing, larvae were poke-wounded (see Materials and Methods), a procedure that produces small and largely symmetrical wounds. Larval whole mounts were immunostained using anti-β-catenin antibodies (Riggleman et al., 1990) at various times after wounding. In unwounded larvae, β-catenin was apparent at cell-cell junctions (compare membrane-GFP and β-catenin staining in Fig. 1A,A′ and Fig. S1A,A′). This signal is specific because expression of an RNAi transgene targeting β-catenin significantly reduced it without reducing an independent membrane label (Fig. S1B′,D). Epidermal expression of an *E-cadherin* RNAi transgene also decreased β-catenin levels at the junctions (Fig. S1C′,D), and vice versa (Fig. S1E,F), indicating that junctional β-catenin depends upon E-cadherin expression as previously reported (Pai et al., 1996). Ten minutes after wounding, β-catenin was still apparent at the interface between wound-edge epidermal cells (Fig. 1B′, arrows). However, β-catenin at most interfaces radial to and immediately proximal to the wound was reduced 1 h (Fig. 1C′, arrowheads) and 2 h (Fig. 1D′, arrowheads) after wounding. After WC was complete at 5 h (Fig. 1E-F), β-catenin staining became diffuse as the epidermal cells presumably remodeled their junctions. Comparison of the ratio of lateral β-catenin levels in first row versus second row cells (Fig. 1G) revealed a clear decrease over the first hour after wounding (Fig. 1H). These results suggest that regulation of β-catenin levels near the wound may be important during epidermal WC.

### The destruction complex regulates junctional β-catenin and is required for wound closure

**Fig. 2.**
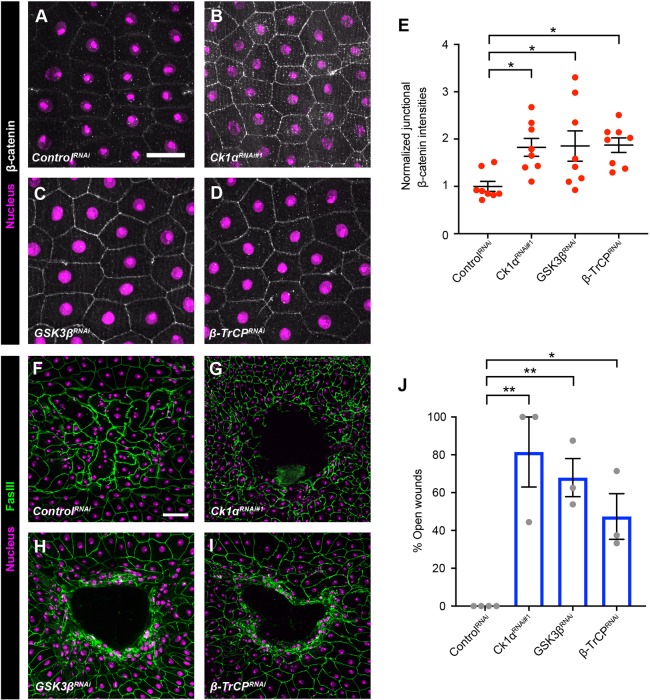
**The destruction complex regulates junctional β-catenin levels and wound closure.** (A-D) Dissected epidermal whole mounts of unwounded third instar larvae expressing *UAS-DsRed2nuc* (nuclei, magenta), *UAS-src-GFP* (cell membranes, green, not shown) and the indicated transgenes via either the *e22c-Gal4* (A,C,D) or *A58-Gal4* drivers (B). Anti-β-catenin antibody staining is in white. (A) *Control^RNAi^*. (B) *Ck1α^RNAi#1^* (*A58-Gal4* used because this line grows slowly with *e22c-Gal4*). (C) *GSK3β^RNAi^*. (D) *β-TrCP^RNAi^*. (E) Quantitation of junctional β-catenin intensity in larvae expressing the different transgenes. Each dot represents an average of the β-catenin signal from five junctions of one larva. Data are mean±s.e.m. *P<0.05 (one-way ANOVA). (F-I) Dissected epidermal whole mounts of pinch-wounded third instar larvae expressing *UAS-DsRed2Nuc* (nuclei, magenta) via the *e22c-Gal4* driver and the indicated RNAi transgenes. Cell boundaries were immunostained using anti-Fasciclin III antibodies (green). Scale bars: 50 μm in A for A-D; 100 μm in F for F-I. (F) *Control^RNAi^*. (G) *Ck1α^RNAi^*. (H) *GSK3β^RNAi^*. (I) *β-TrCP^RNAi^*. (J) Quantitation of the percentage of open wounds in larvae expressing the indicated transgenes via the *e22c-Gal4* driver. Each dot represents one set of *n*≥8 larvae for each genotype. Data are mean±s.e.m. *P<0.05, **P<0.01 (one-way ANOVA).

Because β-catenin levels are decreased near the wound (Fig. 1C′,D′,H), we tested whether the destruction complex regulates β-catenin levels in the larval epidermis. We used immunofluorescence to examine β-catenin levels in the larval epidermis expressing an RNAi transgene targeting *Ck1α*, the key destruction complex kinase that promotes β-catenin degradation. β-Catenin was significantly increased when the larval epidermis expressed *Ck1α^RNAi^* (Fig. 2B,E) compared with a control RNAi transgene (Fig. 2A,E). Intriguingly, β-catenin upregulation was particularly apparent at AJs. The junctional β-catenin (Fig. S2D,E) and E-cadherin (Fig. S2G,H) were also increased in larval epidermis expressing the *Ck1α^RNAi^* transgene via another epidermal driver (*e22c-Gal4*) compared with the control (Fig. S2C,E,F,H).

We next measured whether the increase in β-catenin impacted WC. Expression of *Ck1α^RNAi^* also caused a strong WC defect (Fig. 2G,J). The WC defect was also observed in larvae expressing an RNAi transgene targeting a non-overlapping region of the *Ck1α* sequence (Fig. S2A,B), arguing strongly against RNAi off-target effects. These results suggest that Ck1α negatively regulates β-catenin to facilitate WC. To test whether GSK3β and β-TrCP, other destruction complex components, also regulate β-catenin, we expressed RNAi transgenes targeting them in the larval epidermis. Larvae expressing RNAi transgenes targeting GSK3β or β-TrCP also exhibited a significant increased in β-catenin (Fig. 2C-E) and WC defects (Fig. 2H-J). These results indicate that multiple components of the destruction complex regulate β-catenin levels and epidermal WC.

### The wound closure defect observed with *Ck1α^RNAi^* is β-catenin dependent

**Fig. 3.**
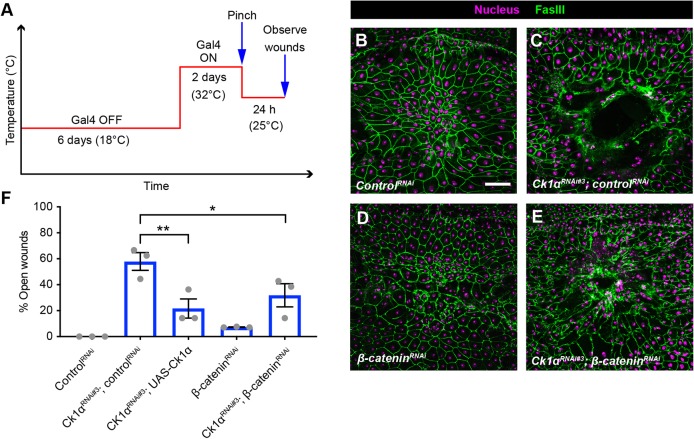
**Silencing β-catenin partially rescues the *Ck1α^RNAi^*-induced wound closure defect.** (A) Schematic of the experimental design/temperature shift regimen for using Gal80^ts^ to inducibly express UAS-dependent transgenes in the larval epidermis. (B-E) Dissected epidermal whole mounts of pinch-wounded third instar larvae expressing *Gal80^ts^* transgene driven by a tubulin promoter, *UAS-DsRed2Nuc* (nuclei, magenta) via the *e22c-Gal4* driver, and the indicated transgenes. Cell boundaries were immunostained using anti-Fasciclin III antibodies (green). (B) *Control^RNAi^*, (C) *UAS-Ck1α^RNAi#3^* and *control^RNAi^,* (D) *UAS-β-catenin^RNAi^*, (E) *Ck1α^RNAi#3^* and *UAS-β-catenin^RNAi^*. Scale bar: 100 μm. (F) Quantitation of the percentage of open wounds in larvae expressing indicated transgenes via the *e22c-Gal4* driver. Each dot represents one set of *n*≥8 for each genotype. Data are mean±s.e.m. *P<0.05, **P<0.01 (one-way ANOVA).

If β-catenin is a crucial downstream target of Ck1α during wound healing, then silencing β-catenin should reduce *Ck1α^RNAi^*-induced WC defects. Continuous epidermal expression of the RNAi line targeting *Ck1α* that can be genetically combined with other transgenes is lethal (RNAi#3 in Fig. S2A). Therefore, we used a temperature-sensitive allele of Gal80 (McGuire et al., 2004) to temporally control transgene expression (see experimental schematic in Fig. 3A). We also verified that the *β-catenin^RNAi^* transgene indeed knocked down β-catenin in this inducible paradigm (Fig. S3D-F). *Ck1α^RNAi^* also increased β-catenin levels in the cytoplasm (compare Fig. S3C with S3B). Junctional E-cadherin was similarly upregulated in the epidermis expressing the *Ck1α^RNAi^* transgene (Fig. S3H,K) compared with the control (Fig. S3G,K), although the trend of increased junctional β-catenin did not reach significance (Fig. S3B,C,F). Importantly, inducible expression of *Ck1α^RNAi^* also caused a strong WC defect that was unaffected by expression of a control RNAi transgene (Fig. 3C,F); this defect was rescued by co-expressing *UAS-Ck1α-HA* (Fig. 3F), further suggesting the WC defect is due to loss of *Ck1α* rather than to off-target effects. Silencing β-catenin by itself did not block WC (Fig. 3D,F) despite a strong reduction in β-catenin (Fig. S3D). However, knocking down β-catenin significantly ameliorated the WC defect observed upon expression of *Ck1α^RNAi^* (Fig. 3E,F). These results suggest that the WC defect observed upon expression of *Ck1α^RNAi^* is dependent on β-catenin levels in the larval epidermis.

### *Ck1α^RNAi^*-induced wound closure defects are related to its functions at the adherens junction rather than to Wnt signaling

As β-catenin is an important downstream transcriptional co-activator of Wnt signaling, we also tested whether Wnt signaling is activated upon wounding. Larvae bearing either *fz3-LacZ* or *6TH-LacZ*, two Wnt signaling reporters (Chang et al., 2008; Sato et al., 1999), were wounded and examined 4 h after wounding. The *fz3-LacZ* reporter was functional as it was activated in the developing wing imaginal discs (Fig. S4A′). However, *fz3-lacZ* was not activated after wounding (compare wounded epidermis in Fig. S4D with unwounded epidermis in Fig. S4A). Similarly, *6TH-LacZ* was also expressed in the wing discs (Fig. S4B′) but not activated in the epidermis after wounding (Fig. S4E versus control in S4B). The JNK reporter, *msn-LacZ*, a positive control, was activated around the wound margin after wounding (Fig. S4C,F) as previously reported (Galko and Krasnow, 2004; Lesch et al., 2010). To test whether Wnt signaling is activated in the epidermis expressing a *Ck1α^RNAi^* transgene, we also examined these two Wnt reporters. The *fz3-LacZ* reporter did not change upon loss of *Ck1α* (Fig. S4I). However, *6TH-LacZ* was activated in the epidermis expressing *Ck1α^RNAi^* transgene (Fig. S4J), suggesting that the Wnt signaling is activated in this condition.

**Fig. 4.**
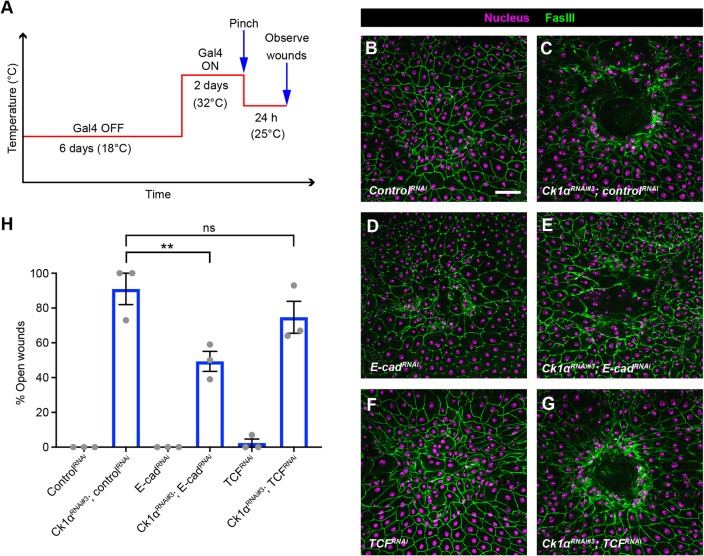
**The epidermal *Ck1α^RNAi^*-induced wound-closure defect is E-cadherin dependent and Wnt signaling independent.** (A) Schematic of the experimental design/temperature shift regimen for using Gal80^ts^ to inducibly express UAS-dependent transgenes in the larval epidermis. (B-G) Dissected epidermal whole mounts of pinch-wounded larvae expressing the *Gal80^ts^* transgene driven by a tubulin promoter, *UAS-DsRed2Nuc* (nuclei, magenta) via the *e22c-Gal4* driver, and the indicated transgenes 24 h after wounding. Cell boundaries were immunostained using anti-Fasciclin III antibodies (green). (B) *Control^RNAi^*, (C) *Ck1α^RNAi#3^* and *control^RNAi^*, (D) *E-cad^RNAi^*, (E) *Ck1α^RNAi#3^* and *E-cad^RNAi^*, (F) *UAS-TCF^RNAi^*, (G) *Ck1α^RNAi^* and *UAS-TCF^RNAi^*. Scale bar: 100 μm. (H) Quantitation of the percentage of open wounds in third instar larvae expressing the indicated transgenes via the *e22c-Gal4* driver. Each dot represents one set of *n*≥8 larvae for each genotype. Data are mean±s.e.m. **P<0.01; ns, not significant (one-way ANOVA).

β-Catenin has roles in both adhesion (Ozawa et al., 1989) and as a transducer of Wnt signaling (Wieschaus et al., 1984). Therefore, we tested which of these two functions impact WC. First, to test genetically whether the *Ck1α^RNAi^*-induced WC defect is related to a role at AJs (as opposed to some other functions of β-catenin), we co-expressed an RNAi transgene targeting E-cadherin, the core component of AJs, to determine whether it could rescue the WC defect observed upon *Ck1α^RNAi^* expression. We again used Gal80^ts^ to temporally control the Gal4 driver activity (see experimental schematic in Fig. 4A). Control larvae closed their wounds 24 h after pinch wounding (Fig. 4B,H), while epidermal expression of *Ck1α^RNAi^* caused a strong WC defect (Fig. 4C,H). As with *β-catenin^RNAi^* (Fig. 3D,F), epidermal expression of an *E-cadherin^RNAi^* transgene by itself did not block WC (Fig. 4D,H). This *E-cadherin^RNAi^* transgene is functional as constitutive expression dramatically reduced junctional E-cadherin (Fig. S1G,H) and β-catenin staining (Fig. S1,C′,D). The *E-cadherin^RNAi^* transgene also worked in this inducible paradigm (Fig. S5D,F for β-catenin and Fig. S5I,K for E-cadherin). Importantly, knocking down E-cadherin significantly reduced *Ck1α^RNAi^*-induced WC defects (Fig. 4E,H), indicating that the WC defect induced by *Ck1α^RNAi^* is related to its regulation of AJs.

To test genetically whether the *Ck1α^RNAi^*-induced WC defect might also be related to a role in Wnt signaling, we co-expressed RNAi transgenes targeting T cell factor (TCF). Epidermal expression of the *TCF^RNAi^* transgene by itself did not block WC (Fig. 4F,H). This *TCF^RNAi^* transgene is functional as its expression in the wing pouch via *nubbin-Gal4* disrupted normal wing development (compare Fig. S5L with S5M), consistent with interference with Wnt signaling. Epidermal expression of a *TCF^RNAi^* transgene did not ameliorate the WC defect induced by *Ck1α^RNAi^* expression (Fig. 4G,H), suggesting that Wnt signaling is not required for the *Ck1α^RNAi^*-induced WC defect.

### β-Catenin overexpression also caused wound closure defects related to its functions at the adherens junction

**Fig. 5.**
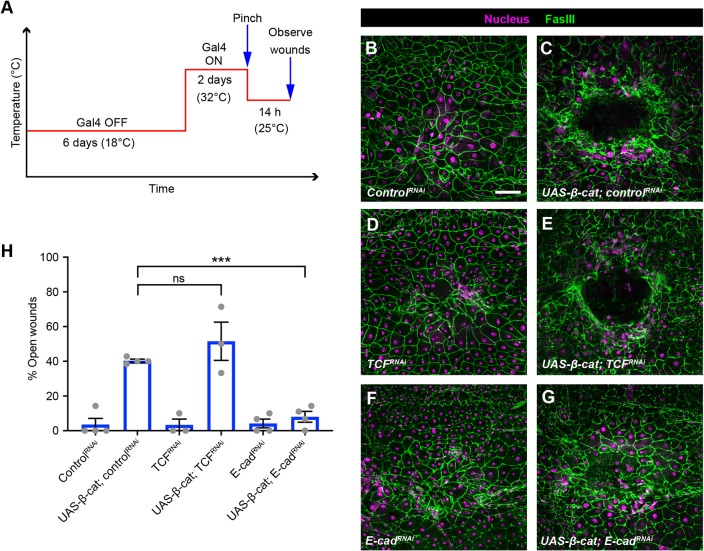
**Epidermal β-catenin overexpression-induced wound closure defect is E-cadherin dependent and Wnt signaling independent.** (A) Schematic of the experimental design/temperature shift regimen for using Gal80^ts^ to inducibly express UAS-dependent transgenes in the larval epidermis. (B-G) Dissected epidermal whole mounts of pinch-wounded larvae expressing the *Gal80^ts^* transgene driven by a tubulin promoter, *UAS-DsRed2Nuc* (nuclei, magenta) via the *e22c-Gal4* driver, and the indicated transgenes 14 h after wounding. Cell boundaries were immunostained using anti-Fasciclin III antibodies (green). (B) *Control^RNAi^*, (C) *UAS-β-catenin* and *control^RNAi^*, (D) *E-cad^RNAi^*, (E) *UAS-β-cat* and *E-cad^RNAi^* (F), *UAS-TCF^RNAi^*, (G) *UAS-β-cat* and *UAS-TCF^RNAi^*. Scale bar: 100 μm. (H) Quantitation of the percentage of open wounds in third instar larvae expressing the indicated transgenes via the *e22c-Gal4* driver. Each dot represents one set of *n*≥8 larvae for each genotype. Data are mean±s.e.m. ***P<0.001; ns, not significant (one-way ANOVA).

An alternative way to test the role of β-catenin during WC is to directly overexpress it in the larval epidermis (see schematic Fig. 5A). Similarly to RNAi transgenes targeting β-catenin-negative regulators (Fig. 2G-J), β-catenin overexpression also blocked WC (Fig. 5C,H) relative to controls (Fig. 5B,H). Because β-catenin is a key component of Wnt signaling, we tested whether β-catenin overexpression activates Wnt signaling in the larval epidermis. The Wnt reporter, *fz3-LacZ* (Fig. S4K), was not activated upon β-catenin overexpression. However, another Wnt reporter, *6TH-LacZ*, was activated (Fig. S4L). If β-catenin-induced Wnt activation is responsible for its WC defect, silencing TCF should rescue the observed WC defect. However, epidermal expression of the *TCF^RNAi^* transgene did not block the β-catenin-induced WC defect (Fig. 5E,H).

To test whether the WC defect caused by β-catenin overexpression is related to its function at AJs, we wounded larvae expressing both *E-cadherin^RNAi^* and *β-catenin* transgenes in the larval epidermis. Interestingly, silencing E-cadherin significantly reduced the β-catenin-induced WC defects (Fig. 5G,H). To test the impact of β-catenin overexpression on E-cadherin, we performed immunostaining using an E-cadherin antibody. Surprisingly, overexpression of β-catenin did not increase E-cadherin (Fig. S6H,K) as with the *Ck1α^RNAi^* transgene (Figs S3H,K and S5H,K). We also examined the sub-cellular locations of β-catenin in these animals and found that although β-catenin is highly expressed in the nuclear and cytoplasmic compartments (Fig. S6C), its levels on the membrane were modest.

### Actin distribution in Ck1α^RNAi^ and β-catenin overexpression

**Fig. 6.**
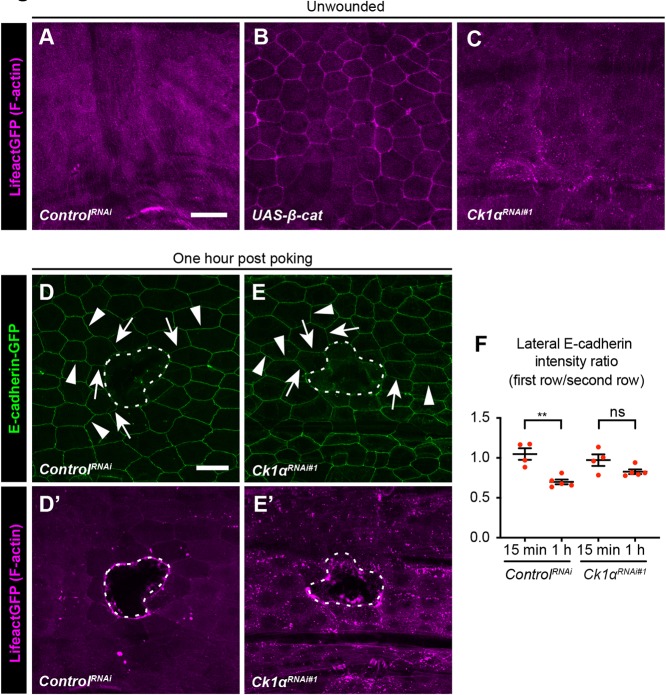
**Actin localization and Ck1α are required for the reduction of lateral E-cadherin on wound-edge epidermal cell membranes.** (A-C) Dissected larval epidermal whole mounts of third instar larvae expressing *UAS-Lifeact-mCherry* (magenta) and the indicated transgenes via the *e22c-Gal4* driver. (D-E′) Live image of larval epidermis expressing *E-cadherin-GFP* and *UAS-LifeAct-mCherry* (magenta) via *e22c-Gal4* driver (control, D,D′) and (*Ck1α*^*RNAi*^, E,E′) 1 h after wounding. (D,E) E-cadherin-GFP, green. (D′-E′) F-actin to visualize the wound margins. Arrows and arrowheads in D,E indicate examples of first-row and second-row junctions, respectively. Scale bars: 50 μm. Dotted white lines indicate wound borders. (F) Quantitation of junctional E-cadherin-GFP ratio in first- and second-row wound-edge epidermal cells. Each dot represents one animal. Data are mean±s.e.m. **P<0.01; ns, not significant (unpaired *t*-test).

Both Ck1*α*^RNAi^ and β-catenin overexpression cause WC defects that are dependent on the levels of E-cadherin. However, these genetic manipulations differ in the levels and cellular distributions of both β-catenin and E-cadherin. Because β-catenin is a key protein that bridges E-cadherin at AJs with the actin cytoskeleton, we checked the distribution of F-actin in these two genotypes. In the control epidermis, the F-actin label LifeAct-mCherry is evenly distributed within epidermal cells and membrane levels are low (Fig. 6A). Strikingly, β-catenin overexpression significantly increased cortical actin (Fig. 6B) while expression of the *Ck1α^RNAi^* transgene did not (Fig. 6C). These results suggest that although both β-catenin overexpression and Ck1α^RNAi^ caused E-cadherin-dependent WC defects, the precise mechanism of their wound closure defects may differ at the level of actin dynamics.

### Ck1α is required for reduction of lateral E-cadherin around the wound

Finally, we tested whether Ck1α is important to reduce the lateral AJs in the wound-edge cells (Fig. 1), which was our initial observation. We examined E-cadherin-GFP levels 1 h after poking. Similar to β-catenin, lateral E-cadherin around the wounds was reduced 1 h after poke wounding (Fig. 6D,D′,F). However, this reduction was ameliorated in the epidermis expressing a *Ck1α^RNAi^* transgene (Fig. 6E-F), indicating that *Ck1α* is involved in local downregulation of lateral junctions near the wound.

## DISCUSSION

In summary, four lines of experimental evidence support the hypothesis that regulation of junctional β-catenin is crucial for epidermal WC in *Drosophila* larvae. First, junctional β-catenin is reduced in wound-edge epidermal cells during WC. Second, RNAi transgenes targeting the β-catenin destruction complex caused upregulation of junctional β-catenin and strong WC defects. Third, the *Ck1α^RNAi^*-induced WC defect was reduced by silencing E-cadherin, indicating that this defect is related to its function at AJs. Finally, *Ck1α^RNAi^* impedes removal of the lateral E-cadherin from lateral junctions radial to the wound. β-Catenin overexpression also caused AJ-dependent WC defects although the mechanism may be distinct given differences in E-cadherin levels, and β-catenin and actin distributions. In summary, regulation of cell-cell interactions through AJs may be a crucial mechanism for coordinating wound-induced cell sheet migration.

In the fly embryo, loss of E-cadherin delays WC (Abreu-Blanco et al., 2013). However, silencing E-cadherin did not block WC in third instar larvae. Similarly, knocking down β-catenin did not cause a WC defect, indicating that the requirement of adherens junctions varies at different developmental stages. Given that the embryonic epidermis has not yet secreted cuticle and that the larval epidermis is tightly adherent to the apical cuticle it secretes (Galko and Krasnow, 2004), some differences in adhesion requirements might be expected. The normal morphology of the E-cadherin and β-catenin-deficient larval epidermis is surprising given how important these proteins are for tissue morphology in development. E-cadherin loss in the mouse causes early embryonic lethality as epithelial tissues fail to form properly (Larue et al., 1994; Riethmacher et al., 1995). Likewise, strong E-cadherin alleles in the fly disrupt early morphogenesis (Oda et al., 1994; Tepass and Hartenstein, 1994). E-cadherin loss in skin so profoundly affects morphology (Tinkle et al., 2008) that wound healing studies are essentially precluded, while β-catenin loss using the same *keratin-14 Cre* driver causes defects in the hair follicle cycle (Huelsken et al., 2001). Conditional knockout of mouse Ck1α causes hyperplasia and pigmentation defects in skin (Chang et al., 2017), and these mice have not been analyzed for wound healing phenotypes.

In the laser-wounded fly embryo, E-cadherin is reduced at the wound perimeter and the lateral interfaces between neighboring wound-edge epidermal cells (Hunter et al., 2015). E-cadherin level is simultaneously increased at the vertices between neighboring wound-edge cells along the wound margin. In both embryos (Hunter et al., 2015) and larvae (this study), ubiquitous epidermal upregulation of AJ components interferes with effective WC. Although Ck1α is required for the local reduction of E-cadherin near wounds, the specific function of wound-edge adherens junction downregulation in embryos and larvae remains technically challenging to test. During collective cell migration in culture, lateral E-cadherin increases tension and slows migration (Suffoletto et al., 2018). A reduction of E-cadherin at the migratory leading edge of mouse wounds, similar to that seen here, has been observed (Hudson et al., 2009). At the functional level, loosening of junctions and reduction of E-cadherin at the leading edge through ephrin signaling appears important for mouse keratinocytes to efficiently move into the wound gap (Nunan et al., 2015). Thus, at least in principle, reduction of junctional components at the lateral interface may reduce tension between wound-edge cells to facilitate efficient WC.

What regulates the redistribution of adherens junctions? E-cadherin genetically interacts with dynamin, an essential regulator of endocytosis, during WC in the fly embryo (Matsubayashi et al., 2015). Notably, endocytosis is also essential for wound healing in the fly embryo (Hunter et al., 2015; Matsubayashi et al., 2015) and E-cadherin reduction around the wound is partially endocytosis dependent (Hunter et al., 2015). Endocytosis may also regulate β-catenin levels in wounded larvae but there is at least one other mechanism, identified here, that seems to do this. That is the activity of the destruction complex – several members of which (Ck1α, GSK3β and β-TrCP), regulate epidermal β-catenin levels and are also required for WC. It is curious that the destruction complex is required for WC but Wnt signaling is not involved. Here, neither knockdown of *TCF* nor β-catenin caused a WC defect, indicating that the transcriptional output of Wnt signaling is not required for WC. The requirement for the destruction complex during WC suggests the possibility that some other signaling pathway required for WC (Baek et al., 2012; Brock et al., 2012; Galko and Krasnow, 2004; Kakanj et al., 2016; Stevens and Page-McCaw, 2012; Tsai et al., 2017; Wu et al., 2009) may regulate β-catenin levels through this complex.

AJs maintain tissue integrity and coordinate cell sheet migration, as these cells are mechanically linked (Pinheiro and Bellaïche, 2018). In embryos, loss of E-cadherin impairs actomyosin ring formation and delays WC (Abreu-Blanco et al., 2013). Interestingly, we did not observe WC defects in larvae lacking epidermal E-cadherin or β-catenin. What accounts for this stage-dependent difference? Occluding (septate) junctions are also required for efficient WC in fly embryos (Carvalho et al., 2018) as are some Integrin complex components in fly larvae (Park et al., 2018). A specific role for occluding junctions in larval WC has not yet been tested. It is also possible that the apical cuticular attachment of larval epidermal cells mechanically stabilizes them in such a way that makes them less sensitive to loss of AJ functions.

During wound healing in fly embryos, gain of E-cadherin reduces wound-edge actin intensity and cell protrusions (Hunter et al., 2015). This suggests an inverse relationship between AJ levels and wound-edge actin polymerization. In embryos and in single-cell wounds in the larval epidermis the formation of an actomyosin ring is important for WC (Abreu-Blanco et al., 2013; Kakanj et al., 2016). Pinch-wounded larvae polymerize actin at the wound edge in a discontinuous fashion (Brock et al., 2012; Tsai et al., 2017) and larval epidermal cells appear to rely more on protrusive migration (Wu et al., 2009) during WC. Here, we observed that larval epidermal cells overexpressing β-catenin have a higher basal level of cortical actin, which may account, in part, for their inability to efficiently close wounds. It will be interesting to test whether gain of β-catenin also impacts wound-edge actin polymerization and cell protrusion activity during epidermal WC in larvae.

## MATERIALS AND METHODS

### Fly genetics

Flies were raised on regular corn meal media. All crosses were raised at 25°C unless indicated. The GAL4/UAS system was used to drive tissue-specific gene expression of transgenes under UAS control (Brand and Perrimon, 1993). For the embryonic and larval epidermis, *e22c-Gal4* was used (Lawrence et al., 1995); for the larval epidermis, *A58-Gal4* was used (Galko and Krasnow, 2004). For fly wing imaginal discs, *nubbin-Gal4* was used (Calleja et al., 1996)*.* For the wound closure assay, we used *e22c-Gal4, UAS-src-GFP, UAS-DsRed2-Nuc* or *A58-Gal4, UAS-src-GFP, UAS-DsRed2Nuc* (Lesch et al., 2010). *UAS-RNAi* lines from the TRiP Bloomington collection were: *JF01792* (*Ck1α^RNAi#1^*), *GL00021* (*Ck1α^RNAi#2^*), *HMS02276* (*Ck1α^RNAi#4^*), *JF02306* (*TCF^RNAi^*), *JF01252* (*β-catenin^RNAi^*), *HMS00693* (*E-cadherin^RNAi^*), *JF01504* (*β-TrCP^RNAi^*) and *JF01355* (*Luciferase^RNAi^*). *UAS-RNAi* lines from the Vienna Drosophila Research Center were: *GD4256* (*Ck1α^RNAi#3^*) and *KK108994* (*GSK3β^RNAi^*)*.* To enhance *GSK3β^RNAi^* and *β-TrCP^RNAi^* knockdown efficiency, larvae were raised at 29°C. Other transgenic lines from Bloomington Stock Center were: #8369, *P{UAS-arm.Exel}2 (UAS-β-catenin)*; *#60584*, *TI{TI}shg^GFP^*; and *#8529*, *P{UAS-lacZ.Exel}2. LacZ* reporters used were: *msn-LacZ* (Spradling et al., 1999); *fz3-LacZ* (Sato et al., 1999); *6TH-LacZ* (Chang et al., 2008). *UAS-LifeAct-mCherry* (Riedl et al., 2008) was used to label actin.

In cases where early expression of UAS transgenes was unhealthy (combination of *UAS-β-catenin* and *UAS-E-cadherin^RNAi^*) or lethal (*UAS-Ck1α^RNAi#3^*), larvae bearing *tubP-gal80^ts^* (McGuire et al., 2003), *e22c-Gal4*, *UAS-src-GFP, UAS-DsRed2Nuc* and toxic UAS transgene were raised for 6 days at 18°C to begin development, shifted to 32°C for 2 days to reach mid-third-instar, and then allowed to recover at 25°C following pinch wounding.

A list of the genotypes of the flies used in each figure is provided in the supplementary Materials and Methods.

### Wounding techniques

Pinch wounding of the mid-third instar larvae was carried out according to our detailed protocol (Burra et al., 2013). Pinch wounds were scored as ‘open’ if the initial wound gap remained after 24 h (or at the indicated timepoint) and as ‘closed’ if a continuous epidermal sheet was observed at the wound site. To calculate the percentage of larvae with open wounds, three sets of *n*≥8 per genotype were pinched and scored for open wounds under a stereo microscope (Leica MZ16FA).

Poke wounding was carried out using a homemade mechanical probe similar to Von Frey filaments, which generate a constant force when bent. These probes were developed by Roger Lopez-Bellido, Patrick Huang and Thomas Wang in the Galko laboratory and are described elsewhere (Lopez-Bellido et al., 2019). To make a poke wound, the mechanical probe (0.004 inch in diameter/2346 kpa pressure applied) was applied to the dorsal side of the mid-third instar larva, abdominal segment 2-4 (A2-4), until the filament bends for ∼1 s. Poked larvae were dissected and stained at various time points after wounding.

### Whole-mount immunofluorescence and *LacZ* staining

The third instar larval epidermis was dissected, fixed and immunostained as detailed previously (Burra et al., 2013). To highlight epidermal cell boundaries (septate junctions), a mouse monoclonal antibody against Fasciclin III was used (1:50; Developmental Studies Hybridoma Bank) (Patel et al., 1987). Rabbit anti-DsRed (Clontech) was used at 1:1000. Mouse-anti-β-catenin was used at 1:75 (Developmental Studies Hybridoma Bank, N2 7A1) (Riggleman et al., 1990). X-Gal staining was performed as described previously (Galko and Krasnow, 2004). X-Gal development at 37°C was 30 min for *msn-LacZ*, overnight for *fz3-LacZ* and 20 min for *6TH-LacZ.*

### Live imaging

Third instar larvae expressing E-cadherin-GFP and *UAS-LifeAct-mCherry* and different UAS transgenes via *e22c-Gal4* were etherized and poke wounded before mounting. 15 min and 1 h after wounding, larvae were etherized, immobilized and immersed in 1:4 (v/v) diethyl ether to halocarbon oil (Sigma, H88098) and covered with glass coverslips. An Olympus FV1000 Confocal microscope and Fluoview software were used to acquire images (Das et al., 2017).

### Imaging analysis

An Olympus FV1000 Confocal microscope and Fluoview software were used to acquire images of the dissected epidermal whole mounts. Leica MZ16FA stereomicroscope with Planapo 1.6× objective and appropriate filters was used for live imaging of epidermal wounds. ImageJ software was used for image processing.

### Quantitation and statistical analysis

Junctional β-catenin levels of unwounded third instar larvae of different genotypes were measured as follows: in ImageJ, a single line was randomly drawn along the anterior-posterior axis across six epidermal cells (cell tri-junctions were avoided). The peak signals at each junction were measured and averaged for each larva. The intensity of different genotypes was normalized to the *UAS-Luciferase^RNAi^* control. Junctional β-catenin signal was greatly reduced in larvae expressing *UAS-β-catenin^RNAi^* or *UAS-E-cadherin^RNAi^* transgenes. Thus, to measure the junctional β-catenin intensities of those larvae, membrane GFP (*UAS-src-GFP*) signal was used to define the cell-cell junctions. The measured junctional β-catenin intensities were further subtracted by the mean intensity of cytoplasmic β-catenin of the same larva. Junctional β-catenin levels in poke-wounded third instar larvae at different time points were measured as follows: in ImageJ, junctional β-catenin intensities of four randomly selected lateral interfaces within the first row or the second row of wound-edge epidermal cells were measured and averaged. The ratios of averaged first row to second row wound-edge cells were compared between different time points. Unpaired two-tailed *t*-test (two groups) or one-way ANOVA (more than two groups, multiple comparisons) were used to test the significance of experiments: ns, not significant; *P<0.05; **P<0.01; ***P<0.001; ****P<0.0001.

## Supplementary Material

Supplementary information
