# Supplementary material for: Casein kinase 1α decreases β-catenin levels at adherens junctions to facilitate wound closure in Drosophila larvae
Source: Development. 2019 Oct 2;146(20):dev175133. doi: 10.1242/dev.175133 (PMC6826034; doi:10.1242/dev.175133)
Supplement: Supplementary information [file develop-146-175133-s1.pdf]

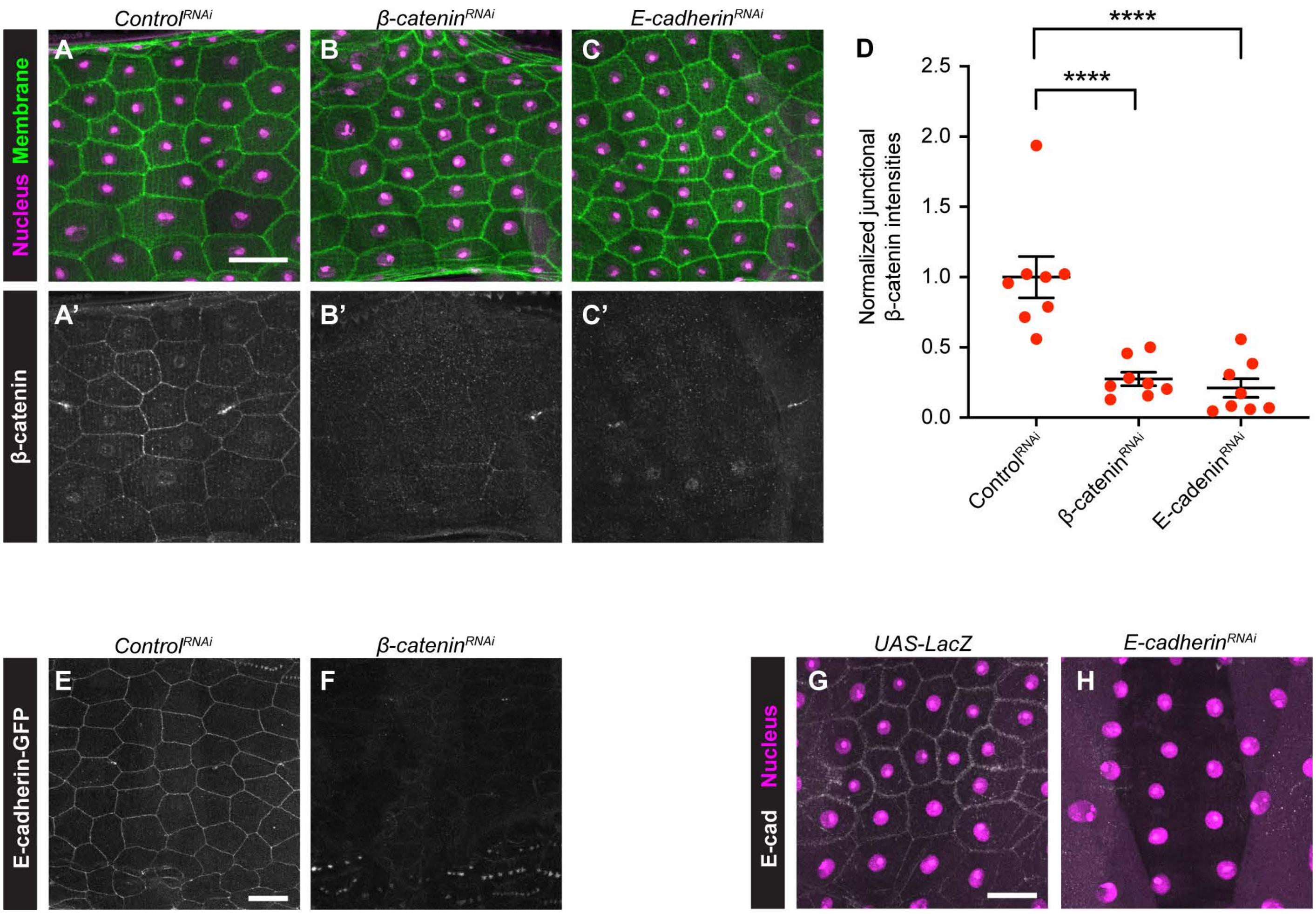

Fig. S1. Validations of β-catenin antibody, *β-catenin*^{RNAi} and *E-cadherin*^{RNAi}.

(A-C') Dissected larval epidermal whole mounts of third instar larvae expressing *UAS-DsRed2nuc* (nuclei, magenta)*,* *UAS-src-GFP* (green) and the indicated transgenes via the *A58-Gal4* driver. (A,A') *Control*^{RNAi}*,* (B,B') *β-catenin*^{RNAi}, (C,C') *E-cadherin*^{RNAi} .(A'-C') Anti-β-catenin, white. Scale bar in (A) is 50 μm for (A-C'). (D) Quantitation of junctional β-catenin intensity in larvae expressing RNAi transgenes targeting components of the adherens junction. Each dot represents the average of the β-catenin signal intensities of five junctions measured within one larva. One-way ANOVA. ****, p<0.0001. (E-F) Dissected larval epidermal whole mounts of third instar larvae expressing *E-cadherin-GFP* and a control *UAS-Luciferase*^{RNAi} transgene (E) or *UAS-β-catenin*^{RNAi} (F) via *e22c-Gal4*. Scale bar in (E) is 50 μm for (E,F). (G-H) Dissected larval epidermal whole mounts of third instar larvae expressing *UAS-DsRed2nuc* (nuclei, magenta) and a control *UAS-LacZ* transgene (G) or *UAS-E-cadherin*^{RNAi} (H) via *A58-Gal4*. Scale bar in (G) is 50 μm for (G,H).

Development • Supplementary information

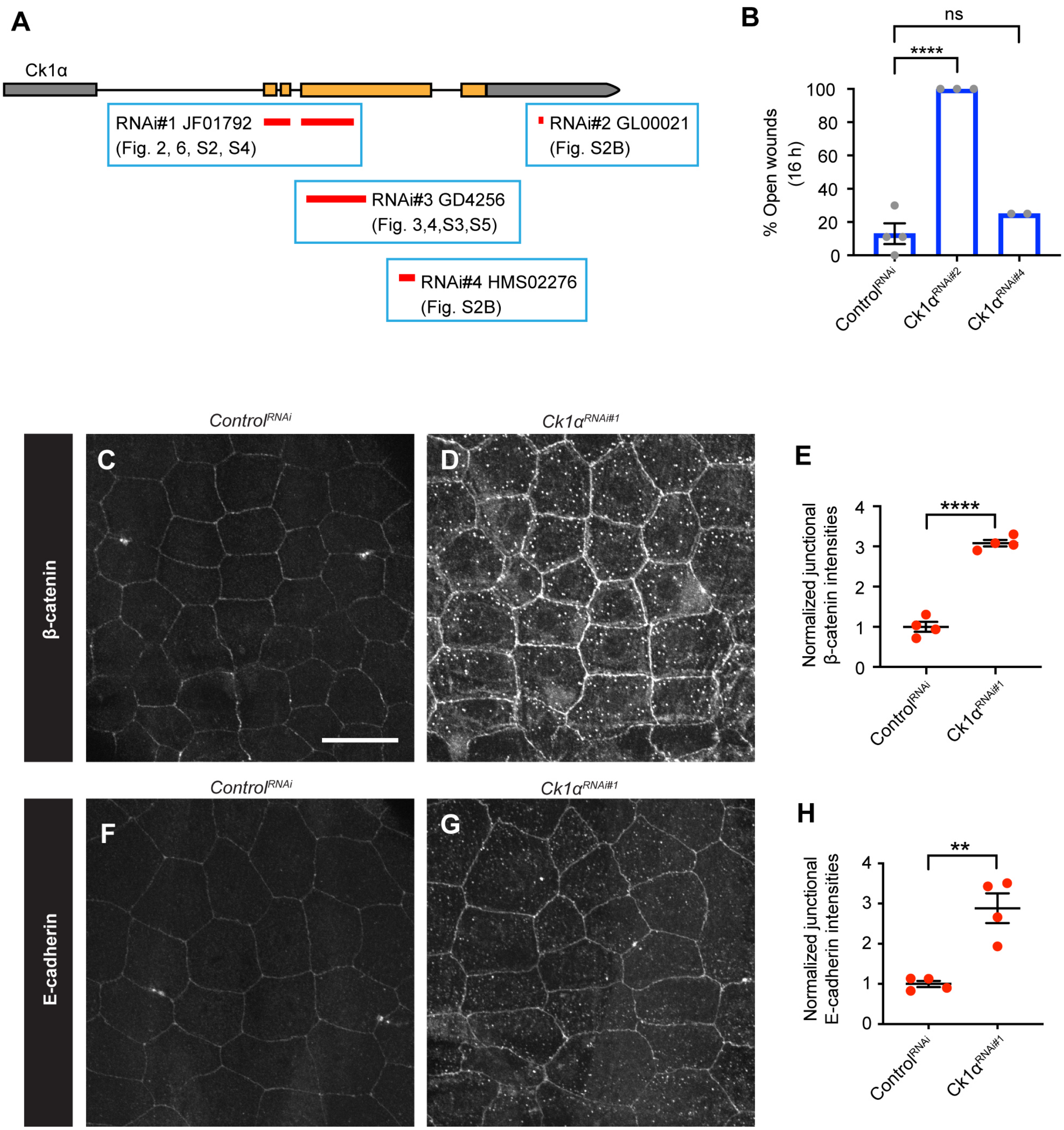
A
Ck1α
RNAi#1 JF01792
(Fig. 2, 6, S2, S4)
RNAi#2 GL00021
(Fig. S2B)
RNAi#3 GD4256
(Fig. 3,4,S3,S5)
RNAi#4 HMS02276
(Fig. S2B)
B
ns
****
% Open wounds
(16 h)
100
80
60
40
20
0
Control^RNAi
Ck1α^RNAi#2
Ck1α^RNAi#4
Control^RNAi
Ck1α^RNAi#1
β-catenin
C
D
E
Normalized junctional
β-catenin intensities
****
4
3
2
1
0
Control^RNAi
Ck1α^RNAi#1
Control^RNAi
Ck1α^RNAi#1
E-cadherin
F
G
H
Normalized junctional
E-cadherin intensities
**
4
3
2
1
0
Control^RNAi
Ck1α^RNAi#1

Fig. S2. Different *Ck1α*^{RNAi} transgenes and their impacts on wound closure and adherens junctions.

(A) Gene regions targeted by *UAS-Ck1α*^{RNAi} transgenes. Continuous epidermal expression of Ck1α^{RNAi} #3 is lethal via *e22c-Gal4* driver. (B) Quantitation of the percent of open wounds in larvae expressing the indicated transgenes via the *e22c-Gal4* driver 16 hours after wounding. Each dot represents one set of n ≥ 8 larvae for each genotype. One-way ANOVA; ****, p<0.0001; ns = not significant. Error bars, standard error of mean. (C-D) Dissected epidermal whole mounts of unwounded third instar larvae expressing *UAS-DsRed2nuc* (nuclei, not shown), *UAS-src-GFP* (cell membranes, not shown) and the indicated transgenes via *e22c-Gal4.* White, anti-β-catenin. Scale bar in (C) is 50 µm for (C,D,F,G). (E) Quantitation of junctional β-catenin intensity in larvae expressing Ck1α^{RNAi} versus control. Each dot represents an average of the β-catenin signal from five junctions of one larva. Unpaired t-test; ****, p<0.0001. (F-G) Dissected epidermal whole mounts of unwounded third instar larvae expressing *UAS-DsRed2nuc* (nuclei, magenta), *UAS-src-GFP* (cell membranes, green, not shown) and the indicated transgenes via *e22c-Gal4.* White, anti-E-cadherin. (H) Quantitation of junctional E-cadherin intensity in larvae expressing Ck1α^{RNAi} versus control. Each dot represents an average of the E-cadherin signal from five junctions of one larva. Unpaired t-test; **, p<0.01.

Development • Supplementary information

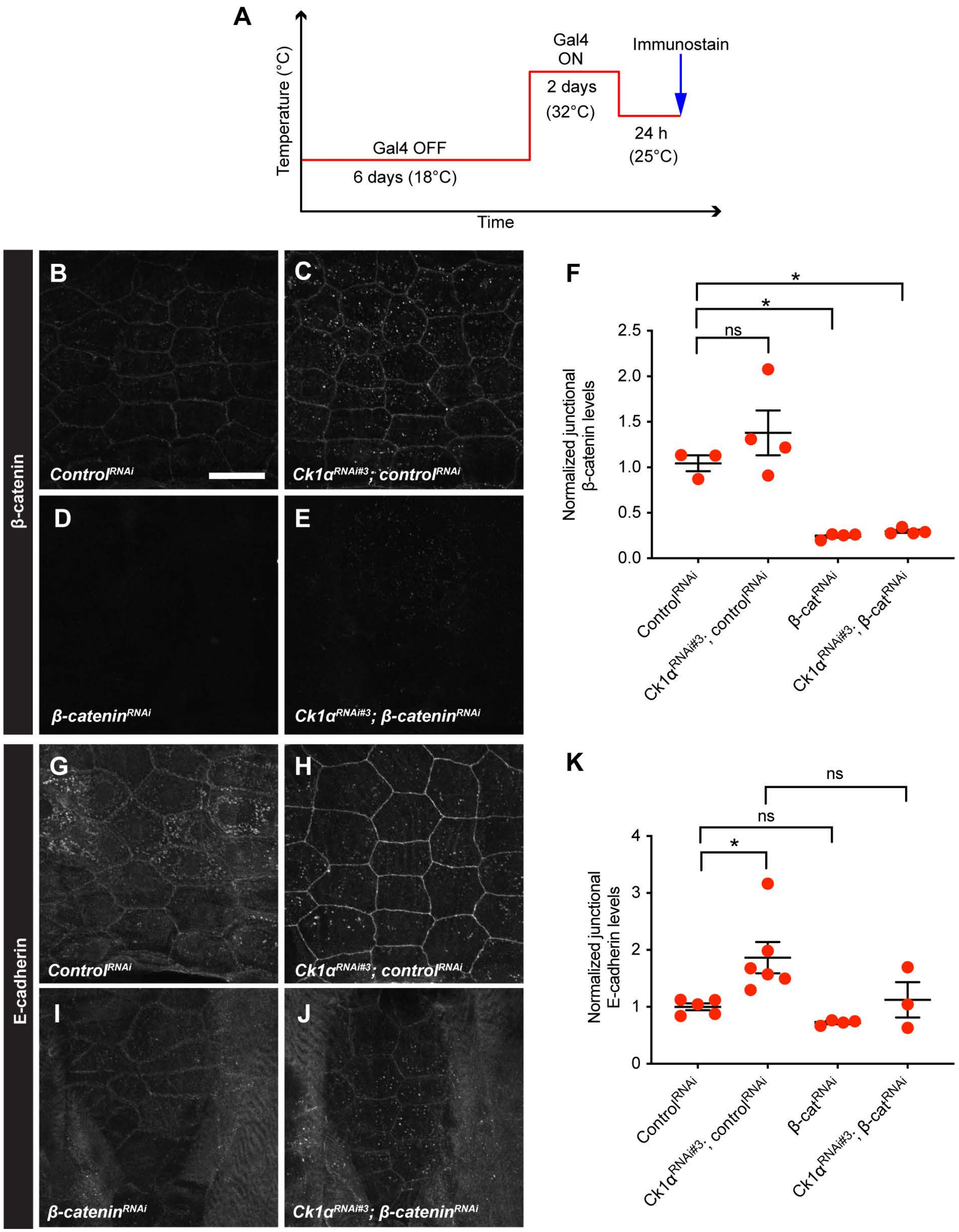
A
Temperature (°C)
Time
Gal4 OFF
6 days (18°C)
Gal4
ON
2 days
(32°C)
24 h
(25°C)
Immunostain
β-catenin
E-cadherin
B
C
D
E
G
H
I
J
Control^RNAi
Ck1α^RNAi#3; control^RNAi
β-catenin^RNAi
Ck1α^RNAi#3; β-catenin^RNAi
F
Normalized junctional
β-catenin levels
K
Normalized junctional
E-cadherin levels
ns
*
Control^RNAi
Ck1α^RNAi#3; control^RNAi
β-cat^RNAi
Ck1α^RNAi#3; β-cat^RNAi

Fig. S3. Epidermal expression of *Ck1α*^{RNAi} transgene increases E-cadherin.

(A) Schematic of the experimental design/temperature shift regimen for using Gal80^{ts} to inducibly express UAS-dependent transgenes in the larval epidermis. (B-E,G-J) Dissected larval epidermal whole mounts of third instar larvae expressing *Gal80*^{ts} transgene driven by tubulin promoter, *UAS-DsRed2nuc* (nuclei, not shown), *UAS-src-GFP* (cell membrane, not shown), and the indicated transgenes via the e22c-*Gal4* driver. (B,G) *Luciferase*^{RNAi}, (C,H) *Ck1α*$^{RNAi\#3}$; *Luciferase*^{RNAi}, (D,I) *β-catenin*^{RNAi} , (E,J) *Ck1α*$^{RNAi\#3}$; *β-catenin*^{RNAi}.(B-E) Anti-β-catenin, white. Scale bar in (B) is 50 µm for (B-E,G-J). (G-J) Anti-E-cadherin, white. Quantitation of junctional β-catenin intensity (F) and E-cadherin intensity (K) in larvae expressing the indicated transgenes. Each dot represents the average of the junctional signal intensities of five junctions measured within one larva. One-way ANOVA. *, p<0.05; ns = not significant.

Development • Supplementary information

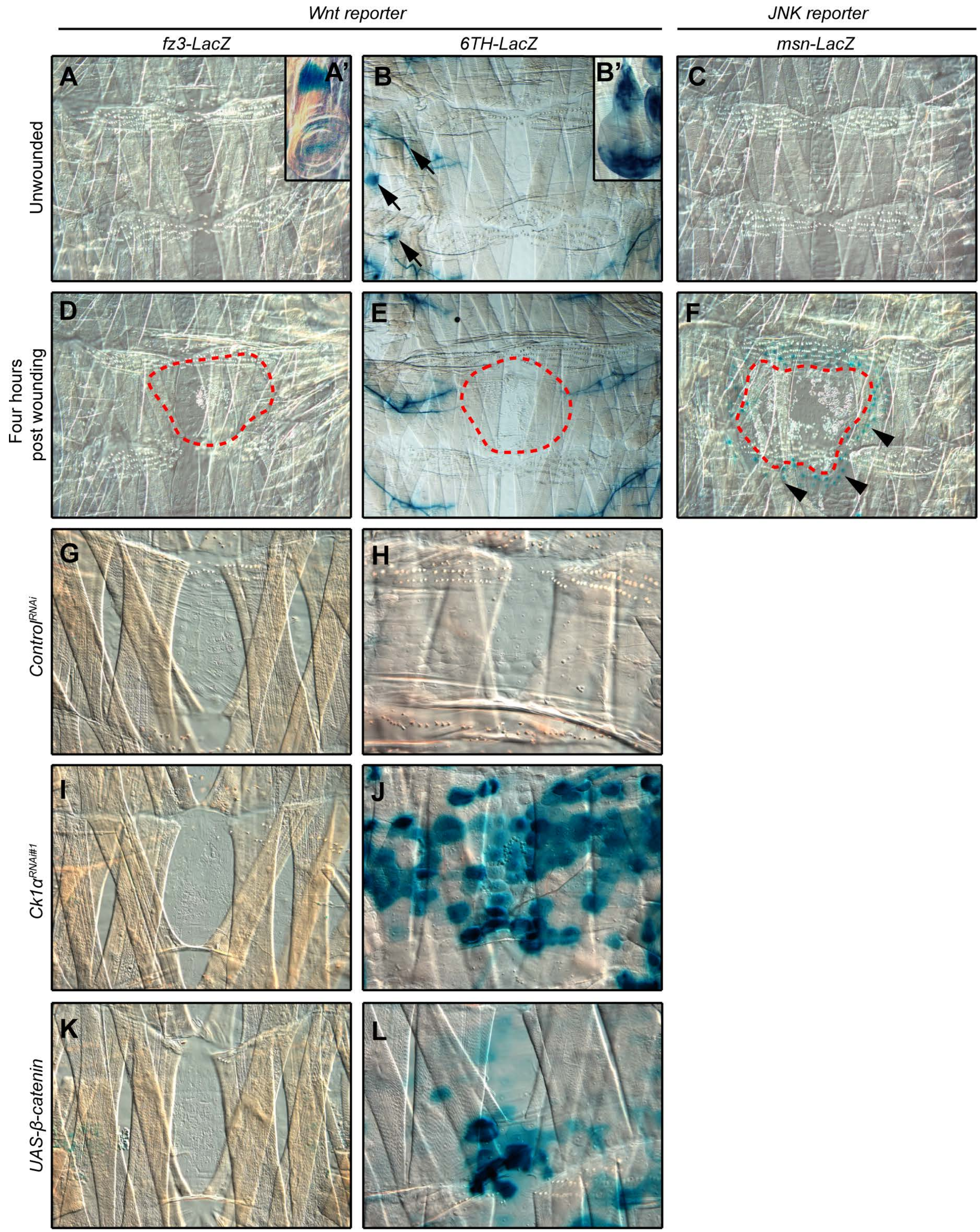

Development • Supplementary information

Fig. S4. Wnt signaling is not activated after wounding but is activated in the larval epidermis lacking Ck1α or expressing β-catenin.

(A-F) X-gal staining (blue) of dissected epidermal whole mounts of unwounded (A-C,G-L) or pinch wounded (D-F) third instar larvae expressing the Wnt reporters *fz3-LacZ* (A,A',D)*, 6TH-lacZ* (B,B',E), or a JNK reporter *msn-lacZ* (C, F). All wounds were examined four hour post-wounding. The wing discs of unwounded larvae expressing the Wnt reporter *fz3-lacZ* (A') or *6TH-LacZ* (B') indicate control staining. Arrows in (B) indicate examples of unrelated signal. Blue dots, LacZ positive nuclei; red dashed line, wound edges. Arrowheads, msn-lacZ positive wound-edge cells. (G-L) X-gal staining (blue) of dissected epidermal whole mounts of unwounded third instar larvae expressing the Wnt reporters *fz3-LacZ* (G, I, K) or *6TH-lacZ* (H, J, L) and the indicated transgenes (left margin) via *e22c-Gal4*.

Development • Supplementary information

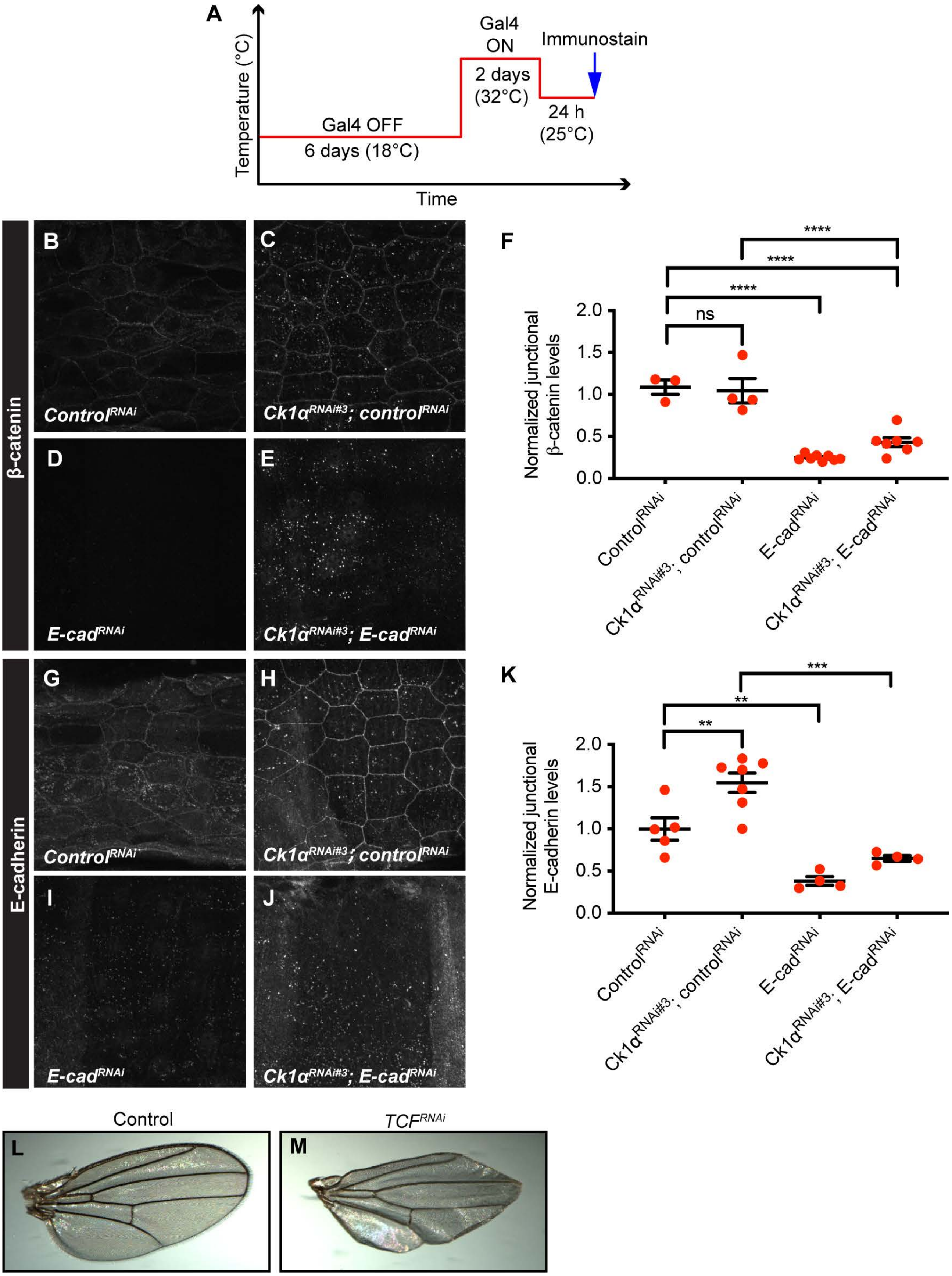
A
Temperature (°C)
Time
Gal4 OFF
6 days (18°C)
Gal4
ON
2 days
(32°C)
24 h
(25°C)
Immunostain
β-catenin
B
Control^RNAi
C
Ck1α^RNAi#3; control^RNAi
D
E-cad^RNAi
E
Ck1α^RNAi#3; E-cad^RNAi
F
Normalized junctional
β-catenin levels
ns
****
****
****
E-cadherin
G
Control^RNAi
H
Ck1α^RNAi#3; control^RNAi
I
E-cad^RNAi
J
Ck1α^RNAi#3; E-cad^RNAi
K
Normalized junctional
E-cadherin levels
**
**
***
Control
TCF^RNAi
L
M

Fig. S5. Validation of conditional *UAS-E-cadherin*^{RNAi} and constitutive *UAS-TCF*^{RNAi} efficacy.

(A) Schematic of the experimental design/temperature shift regimen for using Gal80^{ts} to inducibly express UAS-dependent transgenes in the larval epidermis. (B-E,G-J) Dissected larval epidermal whole mounts of third instar larvae expressing a *Gal80*^{ts} transgene driven by tubulin promoter,*UAS-DsRed2nuc* (nuclei, not shown), *UAS-src-GFP* (not shown), and the indicated transgenes via the e22c-*Gal4* driver. (B,G) *Luciferase*^{RNAi}, (C,H) *Ck1α*$^{RNAi\#3}$; *Luciferase*^{RNAi}, (D,I) *E-cadherin*^{RNAi} , (E,J) *Ck1α*$^{RNAi\#3}$; *E-cadherin*^{RNAi}. (B-E) Anti-β-catenin, white. (G-J) Anti-E-cadherin, white. Scale bar in (B) is 50 µm for (B-E,G-J). Quantitation of junctional β-catenin intensity (F) and E-cadherin intensity (K) in larvae expressing indicated transgenes. Each dot represents the average of the junctional intensities of five junctions measured within one larva. One-way ANOVA. **, $p<0.01$; ***, $p<0.001$; ****, $p<0.0001$. (L,M) Dissected wings of female adult flies expressing the indicated transgenes via a wing pouch driver, *nub-Gal4*, during wing development. The deformed shape of the wing in the *UAS-TCF*^{RNAi} group indicates that this transgene is inhibiting Wnt signaling.

Development • Supplementary information

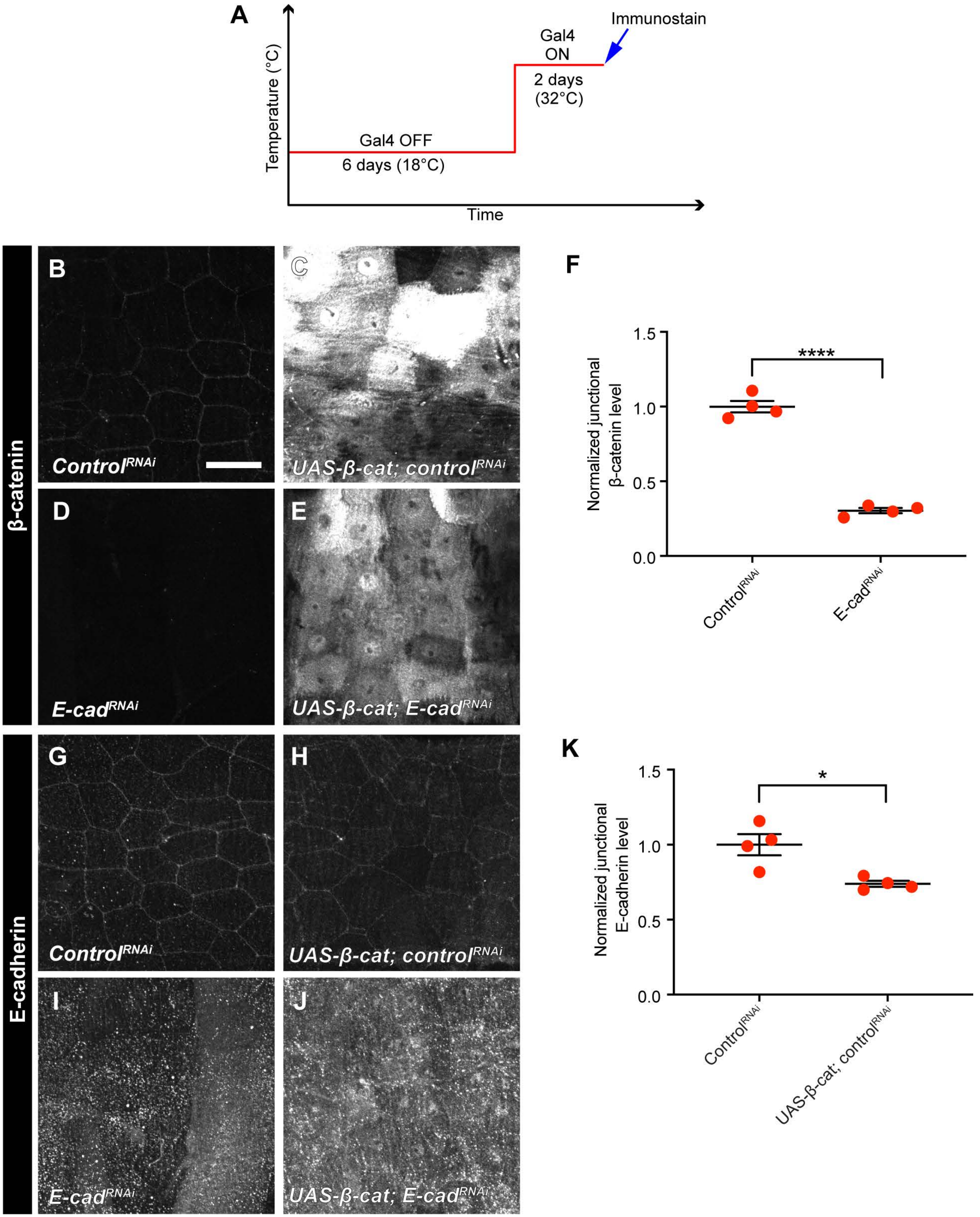
A
Temperature (°C)
Time
Gal4 OFF
6 days (18°C)
Gal4
ON
2 days
(32°C)
Immunostain
β-catenin
B
Control^RNAi
C
UAS-β-cat; control^RNAi
D
E-cad^RNAi
E
UAS-β-cat; E-cad^RNAi
F
Normalized junctional β-catenin level
****
Control^RNAi
E-cad^RNAi
E-cadherin
G
Control^RNAi
H
UAS-β-cat; control^RNAi
I
E-cad^RNAi
J
UAS-β-cat; E-cad^RNAi
K
Normalized junctional E-cadherin level
*
Control^RNAi
UAS-β-cat; control^RNAi

Fig. S6. β-catenin overexpression and *E-cadherin*^{RNAi} effects on β-catenin and E-cadherin expression

(A) Schematic of the experimental design/temperature shift regimen for using Gal80^{ts} to inducibly express UAS-dependent transgenes in the larval epidermis. (B-E,G-J) Dissected larval epidermal whole mounts of third instar larvae expressing a *Gal80*^{ts} transgene driven by tubulin promoter, *UAS-DsRed2nuc* (nuclei, not shown), *UAS-src-GFP* (cell membrane, not shown), and the indicated transgenes via the e22c-*Gal4* driver. (B,G) *Luciferase*^{RNAi}, (C,H) *UAS-β-catenin*; *Luciferase*^{RNAi}, (D,I) *E-cadherin*^{RNAi} , (E,J) *UAS-β-catenin*; *E-cadherin*^{RNAi}. (B-E) Anti-β-catenin, white. Scale bar in (B) is 50 µm for (B-E,G-J). For ease of visualization, panels C and E are imaged at lower laser intensity so as not to be oversaturated. (G-J) Anti-E-cadherin, white. Quantitation of junctional β-catenin intensity (F) and E-cadherin intensity (K) in larvae expressing indicated transgenes. Each dot represents the average of the junctional intensities of five junctions measured within one larva. Unpaired t-test; *, $p<0.05$; ****, $p<0.0001$.

Supplementary Materials and methods

Flies used in this study

Please note the genotype of sex chromosome is simplified. The actual genotypes for the sex chromosome could be mixed, depending on the source RNAi collection, UAS transgenes, and the sex of individual larvae as male and female progeny were pooled together in test populations.

Fig. panels – genotypes tested:

Fig. 1A-E'. *w^{1118};; A58-Gal4, UAS-DsRed2Nuc, UAS-src-GFP/UAS-Luciferase^{RNAi} (JF01355)*

Fig. 1F. *w^{1118}; e22c-Gal4, UAS-LifeAct-mCherry/+; UAS-Luciferase^{RNAi} (JF01355)/+*

Fig. 1H. *w^{1118};; A58-Gal4, UAS-DsRed2Nuc, UAS-src-GFP/UAS-Luciferase^{RNAi} (JF01355)*

Fig. 2A. *w^{1118}; e22c-Gal4, UAS-DsRed2Nuc, UAS-src-GFP/+; UAS-Luciferase^{RNAi} (JF01355)/+*

Fig. 2B. *w^{1118};; A58-Gal4, UAS-DsRed2Nuc, UAS-src-GFP/UAS-Ck1α$^{RNAi\#1}$ (JF01792)*

Fig. 2C. *w^{1118}; e22c-Gal4, UAS-DsRed2Nuc, UAS-src-GFP/UAS-GSK3β^{RNAi} (KK108994)*

Fig. 2D. *w^{1118}; e22c-Gal4, UAS-DsRed2Nuc, UAS-src-GFP/+; UAS-β-TrCP^{RNAi} (JF01504)/+*

Fig. 2E. Same as Fig. 2A-D.

Fig. 2F. *w^{1118}; e22c-Gal4, UAS-DsRed2Nuc, UAS-src-GFP/+; UAS-Luciferase^{RNAi} (JF01355)/+*

Fig. 2G. *w^{1118};; e22c-Gal4, UAS-DsRed2Nuc, UAS-src-GFP/+; UAS-Ck1α$^{RNAi\#1}$ (JF01792)/+*

Fig. 2H. *w^{1118}; e22c-Gal4, UAS-DsRed2Nuc, UAS-src-GFP/UAS-GSK3β^{RNAi} (KK108994)*

Fig. 2I. *w^{1118}; e22c-Gal4, UAS-DsRed2Nuc, UAS-src-GFP/+; UAS-β-TrCP^{RNAi} (JF01504)/+*

Fig. 2J. Same as Fig. 2F-I.

Fig. 3B. *w^{1118}; e22c-Gal4, UAS-DsRed2Nuc, UAS-src-GFP/+; tubP-gal80^{ts}/UAS-Luciferase^{RNAi} (JF01355)*

Development • Supplementary information

Fig. 3C. *w^{1118}; e22c-Gal4, UAS-DsRed2Nuc, UAS-src-GFP/UAS-Ck1α$^{RNAi\#3}$ (GD4256); tubP-gal80^{ts}/UAS-Luciferase^{RNAi} (JF01355)*

Fig. 3D. *w^{1118}; e22c-Gal4, UAS-DsRed2Nuc, UAS-src-GFP/+; tubP-gal80^{ts}/UAS-β-catenin^{RNAi} (JF01252)*

Fig. 3E. *w^{1118}; e22c-Gal4, UAS-DsRed2Nuc, UAS-src-GFP/UAS-Ck1α$^{RNAi\#3}$ (GD4256); tubP-gal80^{ts}/UAS-β-catenin^{RNAi} (JF01252)*

Fig. 3F. Same as Fig. 3B-E.

Fig. 4B. *w^{1118}; e22c-Gal4, UAS-DsRed2Nuc, UAS-src-GFP/+; tubP-gal80^{ts}/UAS-Luciferase^{RNAi} (JF01355)*

Fig. 4C. *w^{1118}; e22c-Gal4, UAS-DsRed2Nuc, UAS-src-GFP/UAS-Ck1α$^{RNAi\#3}$ (GD4256); tubP-gal80^{ts}/UAS-Luciferase^{RNAi} (JF01355)*

Fig. 4D. *w^{1118}; e22c-Gal4, UAS-DsRed2Nuc, UAS-src-GFP/+; tubP-gal80^{ts}/UAS-E-cadherin^{RNAi} (HMS00693)*

Fig. 4E. *w^{1118}; e22c-Gal4, UAS-DsRed2Nuc, UAS-src-GFP/UAS-Ck1α$^{RNAi\#3}$ (GD4256)/; tubP-gal80^{ts}/UAS-E-cadherin^{RNAi} (HMS00693)*

Fig. 4F. *w^{1118}; e22c-Gal4, UAS-DsRed2Nuc, UAS-src-GFP/+; tubP-gal80^{ts}/UAS-TCF^{RNAi} (JF02306)*

Fig. 4G. *w^{1118}; e22c-Gal4, UAS-DsRed2Nuc, UAS-src-GFP/UAS-Ck1α$^{RNAi\#3}$ (GD4256); tubP-gal80^{ts}/UAS-TCF^{RNAi} (JF02306)*

Fig. 4H. Same as Fig. 4B-G.

Fig. 5B. *w^{1118}; e22c-Gal4, UAS-DsRed2Nuc, UAS-src-GFP/+; tubP-gal80^{ts}/UAS-Luciferase^{RNAi} (JF01355)*

Fig. 5C. *w^{1118}; e22c-Gal4, UAS-DsRed2Nuc, UAS-src-GFP/P{UAS-arm.Exel}2 (UAS-β-catenin, BL#8369); tubP-gal80^{ts}/UAS-Luciferase^{RNAi} (JF01355)*

Fig. 5D. *w^{1118}; e22c-Gal4, UAS-DsRed2Nuc, UAS-src-GFP/+; tubP-gal80^{ts}/UAS-TCF^{RNAi} (JF02306)*

Fig. 5E. *w^{1118}; e22c-Gal4, UAS-DsRed2Nuc, UAS-src-GFP/P{UAS-arm.Exel}2 (UAS-β-catenin, BL#8369); tubP-gal80^{ts}/UAS-TCF^{RNAi} (JF02306)*

Development • Supplementary information

Fig. 5F. *w^{1118}; e22c-Gal4, UAS-DsRed2Nuc, UAS-src-GFP/+; tubP-gal80^{ts}/UAS-E-cadherin^{RNAi} (HMS00693)*

Fig. 5G. *w^{1118}; e22c-Gal4, UAS-DsRed2Nuc, UAS-src-GFP/P{UAS-arm.Exel}2 (UAS-β-catenin, BL#8369); tubP-gal80^{ts}/UAS-E-cadherin^{RNAi} (HMS00693)*

Fig. 5H. Same as Fig. 5B-G.

Fig. 6A. *w^{1118}; e22c-Gal4, UAS-LifeAct-mCherry, TI{TI}shg^{GFP} (E-cadherin-GFP, BL#60584)/TI{TI}shg^{GFP} (E-cadherin-GFP, BL#60584); UAS-Luciferase^{RNAi} (JF01355)/+*

Fig. 6B. *w^{1118}; e22c-Gal4, UAS-LifeAct-mCherry, TI{TI}shg^{GFP} (E-cadherin-GFP, BL#60584)/P{UAS-arm.Exel}2 (UAS-β-catenin, BL#8369), TI{TI}shg^{GFP} (E-cadherin-GFP, BL#60584)*

Fig. 6C. *w^{1118}; e22c-Gal4, UAS-LifeAct-mCherry, TI{TI}shg^{GFP} (E-cadherin-GFP, BL#60584)/TI{TI}shg^{GFP} (E-cadherin-GFP, BL#60584); UAS-Ck1α$^{RNAi\#1}$ (JF01792)/+*

Fig. 6D,D'. *w^{1118}; e22c-Gal4, UAS-LifeAct-mCherry, TI{TI}shg^{GFP} (E-cadherin-GFP, BL#60584)/TI{TI}shg^{GFP} (E-cadherin-GFP, BL#60584); UAS-Luciferase^{RNAi} (JF01355)/+*

Fig. 6E,E'. *w^{1118}; e22c-Gal4, UAS-LifeAct-mCherry, TI{TI}shg^{GFP} (E-cadherin-GFP, BL#60584)/TI{TI}shg^{GFP} (E-cadherin-GFP, BL#60584); UAS-Ck1α$^{RNAi\#1}$ (JF01792)/+*

Fig. 6F. Same as Fig. 6D,E.

Fig. S1A,A'. *w^{1118};; A58-Gal4, UAS-DsRed2Nuc, UAS-src-GFP/UAS-Luciferase^{RNAi} (JF01355)*

Fig. S1B,B'. *w^{1118};; A58-Gal4, UAS-DsRed2Nuc, UAS-src-GFP/UAS-β-catenin^{RNAi} (JF01252)*

Fig. S1C,C'. *w^{1118};; A58-Gal4, UAS-DsRed2Nuc, UAS-src-GFP/UAS-E-cadherin^{RNAi} (HMS00693)*

Fig. S1D. Same as Fig. S1A-C'

Fig. S1E. *w^{1118}; e22c-Gal4, TI{TI}shg^{GFP} (E-cadherin-GFP, BL#60584)/+; UAS-Luciferase^{RNAi} (JF01355)/+*

Fig. S1F. *w^{1118}; e22c-Gal4, TI{TI}shg^{GFP} (E-cadherin-GFP, BL#60584)/+; UAS-β-catenin^{RNAi} (JF01252)/+*

Fig. S1G. *w^{1118}; P{UAS-lacZ.Exel}2 (UAS-LacZ, BL#8529)/+; A58-Gal4, UAS-DsRed2Nuc, UAS-src-GFP/+*

Fig. S1H. *w^{1118};; A58-Gal4, UAS-DsRed2Nuc, UAS-src-GFP/ UAS-E-cadherin^{RNAi} (HMS00693)*

Fig. S2B

- *w^{1118}; e22c-Gal4, UAS-DsRed2Nuc, UAS-src-GFP/+; UAS-Luciferase^{RNAi} (JF01355)/+*
- *w^{1118}; e22c-Gal4, UAS-DsRed2Nuc, UAS-src-GFP/+; UAS-Ck1α$^{RNAi\#2}$ (GL00021)/+*
- *w^{1118}; e22c-Gal4, UAS-DsRed2Nuc, UAS-src-GFP/+; UAS-Ck1α$^{RNAi\#4}$ (HMS02276)/+*

Fig. S2C,F. *w^{1118}; e22c-Gal4, UAS-DsRed2Nuc, UAS-src-GFP/+; UAS-Luciferase^{RNAi} (JF01355)/+*

Fig. S2D,G. *w^{1118}; e22c-Gal4, UAS-DsRed2Nuc, UAS-src-GFP/+; UAS-Ck1α$^{RNAi\#1}$ (JF01792)/+*

Fig. S2E. Same as Fig. S2C,D

Fig. S2H. Same as Fig. S2F,G

Fig. S3B,G. *w^{1118}; e22c-Gal4, UAS-DsRed2Nuc, UAS-src-GFP/+; tubP-gal80^{ts}/UAS-Luciferase^{RNAi} (JF01355)*

Fig. S3C,H. *w^{1118}; e22c-Gal4, UAS-DsRed2Nuc, UAS-src-GFP/UAS-Ck1α$^{RNAi\#3}$ (GD4256); tubP-gal80^{ts}/UAS-Luciferase^{RNAi} (JF01355)*

Fig. S3D,I. *w^{1118}; e22c-Gal4, UAS-DsRed2Nuc, UAS-src-GFP/+; tubP-gal80^{ts}/UAS-β-catenin^{RNAi} (JF01252)*

Fig. S3E,J. *w^{1118}; e22c-Gal4, UAS-DsRed2Nuc, UAS-src-GFP/UAS-Ck1α$^{RNAi\#3}$ (GD4256); tubP-gal80^{ts}/UAS-β-catenin^{RNAi} (JF01252)*

Fig. S3F. Same as Fig. S3B-E

Fig. S3K. Same as Fig. S3G-J

Fig. S4A,D. *w^{1118};; fz3-LacZ/+*

Fig. S4B,E. *w^{1118}; 6TH-LacZ/+*

Fig. S4C,F. *w^{1118};; A58-Gal4, msn-LacZ/+*

Fig. S4G. *w^{1118}; e22c-Gal4, UAS-DsRed2Nuc, UAS-src-GFP/+; fz3-LacZ/UAS-Luciferase^{RNAi} (JF01355)*

Fig. S4H. *w^{1118}; e22c-Gal4, UAS-DsRed2Nuc, UAS-src-GFP/+; 6TH-LacZ/UAS-Luciferase^{RNAi} (JF01355)*

Fig. S4I. *w^{1118}; e22c-Gal4, UAS-DsRed2Nuc, UAS-src-GFP/+; fz3-LacZ/UAS-Ck1α$^{RNAi\#1}$ (JF01792)*

Fig. S4J. *w^{1118}; e22c-Gal4, UAS-DsRed2Nuc, UAS-src-GFP/+; 6TH-LacZ/ UAS-Ck1α$^{RNAi\#1}$ (JF01792)*

Fig. S4K. *w^{1118}; e22c-Gal4, UAS-DsRed2Nuc, UAS-src-GFP/ P{UAS-arm.Exel}2 (UAS-β-catenin, BL#8369); fz3-LacZ/+*

Fig. S4L. *w^{1118}; e22c-Gal4, UAS-DsRed2Nuc, UAS-src-GFP/P{UAS-arm.Exel}2 (UAS-β-catenin, BL#8369); 6TH-LacZ/+*

Fig. S5B,G. *w^{1118}; e22c-Gal4, UAS-DsRed2Nuc, UAS-src-GFP/+; tubP-gal80^{ts}/UAS-Luciferase^{RNAi} (JF01355)*

Fig. S5C,H. *w^{1118}; e22c-Gal4, UAS-DsRed2Nuc, UAS-src-GFP/UAS-Ck1α$^{RNAi\#3}$ (GD4256); tubP-gal80^{ts}/UAS-Luciferase^{RNAi} (JF01355)*

Fig. S5D,I. *w^{1118}; e22c-Gal4, UAS-DsRed2Nuc, UAS-src-GFP/+; tubP-gal80^{ts}/UAS-E-cadherin^{RNAi} (HMS00693)*

Fig. S5E,J. *w^{1118}; e22c-Gal4, UAS-DsRed2Nuc, UAS-src-GFP/UAS-Ck1α$^{RNAi\#3}$ (GD4256)/; tubP-gal80^{ts}/UAS-E-cadherin^{RNAi} (HMS00693)*

Fig. S5F. Same as Fig. S5B-E

Fig. S5K. Same as Fig. S5G-J

Fig. S5L. *w^{1118}; nubbin-Gal4/+; UAS-Luciferase^{RNAi} (JF01355)/+*

Fig. S5M. *w^{1118}; nubbin-Gal4/+; UAS-TCF^{RNAi} (JF02306)/+*

Fig. S6B,G. *w^{1118}; e22c-Gal4, UAS-DsRed2Nuc, UAS-src-GFP/+; tubP-gal80^{ts}/UAS-Luciferase^{RNAi} (JF01355)*

Development • Supplementary information

Fig. S6C,H. *w^{1118}; e22c-Gal4, UAS-DsRed2Nuc, UAS-src-GFP/P{UAS-arm.Exel}2 (UAS-β-catenin, BL#8369); tubP-gal80^{ts}/UAS-Luciferase^{RNAi} (JF01355)*

Fig. S6D,I. *w^{1118}; e22c-Gal4, UAS-DsRed2Nuc, UAS-src-GFP/+; tubP-gal80^{ts}/UAS-E-cadherin^{RNAi} (HMS00693)*

Fig. S6E,J. *w^{1118}; e22c-Gal4, UAS-DsRed2Nuc, UAS-src-GFP/P{UAS-arm.Exel}2 (UAS-β-catenin, BL#8369); tubP-gal80^{ts}/UAS-E-cadherin^{RNAi} (HMS00693)*

Fig. S6F. Same as Fig. S6B,D.

Fig. S6K. Same as Fig. S6G,H
